# Medical News

**Published:** 1856-06

**Authors:** 


### MEDICAL NEWS.

Dr. Mütter, influenced, we regret to say, by his failing health, has resigned the Chair of Surgery, so long and ably occupied by him in the Jefferson Medical College.

As a teacher and lecturer Dr. Mütter had few equals, and his resignation as well as the cause of it will be universally and very sincerely regretted by the profession.

In consequence of the above resignation, the following resolutions were passed at a meeting of the Board of Trustees of Jefferson Medical College, held May 24th.

*Resolved*, That the Board of Trustees have received with deep regret the letter of Professor Thomas D. Mütter, tendering the resignation of the Chair of Surgery, in the Jefferson Medical College, and give their assent to his request solely on the ground of his impaired health, which, in the opinion of Professor Mütter, renders him unable to discharge its duties.

*Resolved*, That the Board sympathise with Professor Mütter, in the causes that have led to his resignation, and fervently hope that a cessation from the arduous duties in which he has been engaged, may lead to a restoration of his health, and to a long career of eminent usefulness.

*Resolved*, That, as a mark of the high estimation in which the Board of Trustees hold the distinguished services of Professor Mütter, during his long connection with the Institution, they hereby confer on him the honorary distinction of *Emeritus Professor of Surgery* in the Jefferson Medical College.

At a meeting of the Faculty of the Jefferson Medical College, held May 22nd, the following resolutions were passed:

*Resolved*, That whilst the Faculty of Jefferson Medical College have been apprehensive from the correspondence which has lately taken place between them and Professor Mutter, that he might feel impelled to withdraw from his connection with the College, they indulged the hope, that he might still determine to continue in his sphere of eminent usefulness.

*Resolved*, That the intelligence of the resignation of Professor Mutter is, to the Faculty, a matter of the deepest sorrow. As a member of the Faculty through a long series of years, his conrse has been most satisfactory to his Colleagues; and as a teacher he has been as popular as he was distinguished.

*Resolved*, That the Faculty part with him with the most profound regret; and with heart-felt wishes, that his health may be fully restored, and that his future career may be one of unmixed happiness.

We understand that it is the intention of Dr. Mütter to present the College of Physicians, of Philadelphia, his very valuable, extensive and magnificent museum, consisting of calculi, bones, wet preparations, casts, models and drawings.

It is also the intention of Dr. Mütter to endow the College with the sum of thirty thousand dollars, part of the interest of which sum will be devoted to maintaining the museum presented by him, in good order, and adding to it yearly new preparations. In addition to these, a Lectureship on Surgical Topics will be founded—the lecturer to be appointed every third year, so as to afford the different members of the College the opportunity of lecturing, if so disposed.

---

We shall publish the Proceedings of the State Medical Society, which has just terminated its Session, in our next number. Dr. R. La Roche was elected the President for the ensuing year.

---

Dr. S. D. Gross, Professor of the Principles and Practice of Surgery, in the University of Louisville, has been unanimously elected by the Trustees of the Jefferson Medical College to fill the Chair made vacant by the resignation of Dr. Mutter.

Dr. Gross' reputation is so widely extended, and his ability so well known to the profession, that we shall make no comments upon them. His appointment can not fail to give general satisfaction.

---

Alexis St. Martin, the celebrated subject of Dr. Beaumont's experiments, recently visited our city under the care of Dr. Bunting, of Canada. During his stay here, a series of experiments were performed upon him by Dr. F. G. Smith, the results of which will be given in our next number.

---

The Fourteenth Annual Report of the English Registrar General, taken in connexion with the Census Report, shows the influence of certain occupations on mortality to be very different from what was previously supposed.

Still, there are certain occupations sufficiently defined to obviate all danger of their being confounded, and whose rate of mortality can now be recorded with certainty. We give these classes at the decennial period, ranging from 45 to 55, which shows the advancing rate of mortality in twelve occupations.

1. *Farmers.*—Of the twelve classes under consideration, Farmers are the longest livers, their rate of mortality being not quite 12 in 1000 (11·99). The number of English farmers of all ages in 1851, including 2429 graziers, was 225,747, of whom there were 53,608 between the age of 45 and 55. In that year, the total number of deaths among farmers of all ages was 6426, very much below the numbers which would have been registered had these individuals been engaged in other pursuits. These facts prove that the pure air, the daily exercise, the substantial fare, and the other aids to health enjoyed by this substantial class, considerably modify the influence of unfavorable weather, bad seasons, open ports, peculiar burdens on land, and all other ruinous things which farmer's friends have been accustomed to depict in such gloomy colors.

2. Shoemakers hold the next place to farmers, their rate of mortality between 45 and 55 being 15.03 in 1000. They are followed by 3. Weavers, 15.37 ; 4. Grocers, 15.79 ; 5. Blacksmiths 16.51 ; 6. Carpenters, 16.57 ; 7. Tailors, 16.74 ; 8. Laborers 17.30. As will be seen on inspection, there is among these seven occupations a gradual increase in the rate of mortality, which, considering their great diversity, is quite remarkable. The near approach of these occupations to each other in the scale of mortality, arises from the circumstance that they have peculiar dangers which tend to counterbalance each other. Thus it is to be noticed, that the tailor is not exposed to the explosions which are fatal to the miner, and the laborer has exercise which is denied to the tailor.

Ascending the scale of danger we pass to—9. Miners, 20.15 in 1000 ; 10. Bakers, 21.21 ; 11. Butchers, 23.10 ; 12. Innkeepers, 28.34."

" A great disparity is observable in passing from laborers into the class of miners, telling a tale of dangers, many of which result from criminal neglect. Between laborers and the last four classes in this table there is a most remarkable hiatus. In the classes previously noticed, the difference in no case is more than one in a thousand, and in some instances less. Here the difference begins with three, and mounts up to nine in a thousand."

The returns show that the highest rates of mortality are found among the Butchers (23·10 in 1000), and the class of Innkeepers and licensed victuallers (28·34 in 1000).

The extraordinary mortality of butchers is a fact for which we are indebted wholly to the last Census. The "red-injected face" of the butcher, has produced a wrong idea as to the healthy nature of his occupation. This idea is now corrected by scientific induction, and proper sanatory means will overcome the evil thus brought to light. To quote the significant remarks in the report conveying this fact, here is an important problem for solution : "On what does the great mortality of the butcher depend ? On his diet, into which too much animal food, and too little fruit and vegetables enter ? on his drinking to excess ? on his exposure to heat and cold ? or, which is probably the most powerful cause, on the elements of decaying matter by which he is surrounded in his slaughter-house and its vicinity ?"

If the rate of mortality among innkeepers, licensed victuallers, and beer-shop keepers should be seized with avidity by the advocates of teetotalism, they must not be forbidden its use ; at the same time they must be reminded, that " many highly respectable men of this class lead regular lives, and are of steady habits ; but others exposed by their business to unusual temptations, live intemperately and enjoy less quiet at night than the rest of the community. They are exposed also to zymotic diseases, by intercourse with large numbers of people."

Startling and painful as are these disclosures, they cannot be too widely published. They have a practical value among those who deal with the averages of life, for commercial or benevolent purposes ; while, to those more specially concerned, they show the necessity, for their own safety, of employing the measures by which unnecessary disease and premature death may be obviated.

Startling Mesmeric Effects.—The Johnson (Mich) Citizen, of last week, says: " Dr. Samuel P. Hart was tried in Circuit Court, Judge Johnson, for committing a rape on the person of Miss Caroline Church. He was convicted, and sentenced to ten years' imprisonment in the State Prison. It appears from the evidence that Miss Church was being magnetized by the defendant, for a paralysis of one limb and an arm. Some nine months subsequent she was delivered of a child. She swore that she did not know whose child it was; that she never had intercourse with any man to her knowledge; and that she did not know her situation until confined. The parents of the girl swore that young men did not visit her, and that the defendant had ample opportunity to commit the offence. The people also introduced two gentlemen who have been in the habit of magnetizing so as to render the patient unconscious. The trial lasted two days. A. Blair appeared for the people, and S. C. Wood for the defence.

It must be pleasant to live in a country where men are thus convicted of infamous and atrocious crimes without a particle of proof, but simply because a jury did not know who did commit them if the party on trial did not. There does not seem in this case to have been the least evidence of the guilt of Dr. Hart, nor any attempt to prove anything more than he *might* have committed the offence if he had been so disposed.

